# The Question of Local Anæsthetic Nostrums

**Published:** 1893-09

**Authors:** Edward C. Kirk

**Affiliations:** Philadelphia, Pa.


ARTICLE II.

### THE QUESTION OF LOCAL ANÆSTHETIC NOSTRUMS.

BY EDWARD C. KIRK, D. D. S., PHILADELPHIA, PA.

Read before the First District Dental Society, State of New York, March 14, 1893.

The discovery of the local anæsthetic properties of cocain, the active principle of the Erythroxylon coca, has among other things resulted in the production of a crop of nostrums, which are advertised and put upon the market for specific use by injection into the gum-tissue to render the operation of tooth-extraction painless.

It is noteworthy that this method of producing local anæsthesia was not practiced—in fact, was unknown—until after the discovery of the anæsthetic properties of cocain and its salts. Since then, however, the number of nostrums offered to the dental profession for this purpose has rapidly increased, until at present their name is legion, and they continue to multiply, seemingly in a geometrical ratio, each and all claiming to be the "best," "safest," and altogether "most satisfactory,"—and apparently the end is not yet. Such a multiplicity of preparations, each recommended as efficient and reliable, none lacking in numbers of enthusiastic advocates, whose laudatory testimonials are appended to the advertising circulars accompanying the preparations, would appear to indicate a widespread use of these nostrums by dental practitioners. Private letters of inquiry constantly received by your essayist also tend to confirm this view, so that the question of the status of this class of preparations and the attitude of the dental profession toward their use is one that seems to need definition,—to which end I shall ask your consideration of the results of an investigation which I have undertaken with regard to this question, in the hope that it may lead to some well-defined action on your part with reference to the attitude of this society, if not the whole profession, respecting these preparations.

For the sake of clearness, let us discuss the question under two divisions: First, that of nostrums in general; and second, this class of nostrums in particular. It is well to distinguish at the outset the difference between a patented medicine formula and one the composition and ingredients of which are kept secret. The term nostrum has been used to designate both, but Webster restricts it to secret formulæ. The term *arcanum* is used by Dunglison to describe secret formulæ especially, and this word is defined as "any recipe or preparation reputed to possess great efficacy, whose composition is kept secret."

The distinction between patented formulæ and those which are kept secret is of importance, inasmuch as the patenting of a formula is in some degree equivalent to making its composition public, as the records of these patent formulæ are accessible to all who care to investigate them. The formulæ of secret preparations, on the contrary, are the sole property of the proprietors or their agents.

A certain number of dental practitioners, forming an organized body and having in mind the conservation of the best interests of the profession, have formulated certain rules for their guidance, which rules are known as the Code of Ethics, and adherence to these is made the measure of reputability which in their view distinguishes those practitioners who are animated by right motives toward their professional brethren from those who are not so animated. The ethical principle upon which the code rests is that which animates all communities whose moral growth has progressed to a point when it is possible for their members to conceive the maxim, "Do unto others as ye would that they should do unto you," and live up to it. That the evolution of this maxim in a community does actually represent a higher and better status of society is perfectly demonstrable objectively; therefore we are warranted in asserting that those who are animated by this principle represent the better element in any society, the dental profession included.

With respect to the use of nostrums, the Code of Ethics of the American Dental Association, in Article II, section 3, under head of "Maintaining Professional Character," says, "It is unprofessional to circulate or recommend nostrums, or to perform any other similar acts," which means that the moral sense of the better element in dentistry is against the use of these preparations. In his annual address in 1892, the president of the Pennsylvania State Dental Society, at its Cresson meeting, expressed his views upon the subject as follows: "If we are professional at all, it is entirely inconsistent with the pretension, that any one should secure control of procedures or the use of appliances for his own exclusive benefit, and unworthy those who are under the moral obligation to fulfill the maxim, 'Freely as ye have received, freely give.' The endeavor to impose upon the profession by dispensing, for gain, secret formulæ of any of the preparations or materials we use is a still more reprehensible practice, and the use of such should be excluded from any and every society. To effectually stamp out this evil there appears only one means of action, which is for each practitioner to refuse to use any preparation the ingrediency and the proportions of which are unknown to him."

During the ensuing discussion of the address, Dr. J. Allen Osmun said, "There is no discovery or improvement in the practice of dentistry that would benefit the profession or their patients that the dentist who possesses it is not under bounden obligation to give to the profession. He owes it to them."

Under the title, "The Right and Wrong of It," the Philadelphia *Medical News*, December 24, 1892, quotes the following from the *American Lancet:* The *Practitioners' Monthly* says, 'It is not wrong to use a proprietary medicine because the code says so or because some men think so. It is wrong because it damages both medicine and pharmacy. It is wrong because it retards progress, sacrifices the good of the mass for the profit of the individual. The code of men's thoughts are of value only as they represent facts.' " And again, the *Journal of the American Medical Association*, October 8, 1892, prints the following under the title, "The Falsity of Nostrum Dealing:" "*The Positive Medicator* of June has formulated the following conclusions as the result of a very full knowledge of the ways and means of the patent-medicine vendors:

"1. They claim to be specifics, which they are not.

"2. The consumer pays an excessive price for a secret preparation when, were its formula known, the same preparation could be prepared and sold by his druggist at a reasonable figure.

"3. Simple remedies are clouded in secrecy, and sold as valuable new discoveries, which they are not.

"4. Nostrums interfere with legitimate pharmacy, and being sold by dry-goods bazaars, rob the pharmacist of his right alone to compound simple remedies for simple ailments.

"5. Their selling value depends not upon their merit, but entirely upon their being pushed by advertising, which advertising in the end the consumer has to pay for.

"6. There is no question but that the manner of advertising many nostrums is injurious to the public good. Ignorant people with slight illnesses are made to believe that they are in dangerous conditions, and frightened into buying and consuming stuff which may not at all be subject to their cases, and which they use at an excessive cost to their pocket-books and general health," and then adds: "From the above statement it will be seen that it is practically impossible for the nostrum-maker to say a true word in any of his published statements. One ingenious circulator has figured out the cost of production of a dollar bottle of advertised medicine, and he has found that the utmost of drug-value that the manufacturer can afford to deliver to the sick person is ten cents. In other words, ninety cents out of the dollar paid by the consumer are necessarily absorbed by the proprietor, the wholesale druggist, the local dealer, and the newspapers and other channels of spreading the lying claims of the nostrum, before the deluded patient receives his dime's worth of noxious liquid. And doubtless it is better for the patient that he gets that small return for his dollar; the less he gets for his money the better off he will be."

Sufficient has perhaps been said to place before you a reasonable clear presentation of the reasons why the ethical sense of both medical and dental practitioners of the better class is antagonistic to the use of nostrums in general.

Let us now examine the question with respect to the special class of nostrums advertised for the painless extraction of teeth, whose proprietors are flooding the country with laudatory circulars, each one claiming to be the best; new ones appearing in such rapid succession that they seem to "breed like wolves in a famine-stricken land."

The fact that this class of preparations has made its appearance *since* the discovery of the local anæsthetic properties of cocain and its salts, and the close resemblance to cocain anæsthesia of the anæsthetic action of all these nostrums which I have examined, led me to suspect that they depended on cocain in each instance for their activity, if they really had any,—especially as until quite recently no other drug was known to possess the obtunding property in anything like the kind or degree belonging to cocain. I therefore obtained samples of ten of the various preparations most extensively known and advertised as local anæsthetics for painless extraction of teeth, taking especial care in each instance to secure original unbroken packages from the manufacturer or his agent. These I had analyzed by the professor of chemistry at the Philadelphia College of Pharmacy, Professor Samuel P. Sadtler, Ph. D., a chemist of large reputation and experience, and of unquestioned ability, especially in this field of work. His communication of results is as follows:

PHILADELPHIA, February 2, 1893.

DR. E. C. KIRK:

*Dear Sir*,—In December last, I received a package containing samples of so-called "local anæsthetics," with a request that I give them a careful chemical analysis, particularly noting the percentage of cocain salt which might be present.

The list of designations of these ten samples, with a description of their appearance, is as follows:

1. *Dickson's Improved Anæsthetic.* For painless extraction of teeth From the Dickson Manufacturing Company, Sharon, Pa. Colorless liquid, with odor of carbolic acid.

2. *Arophene.* A local anæsthetic for the painless extraction of teeth. From the Arophene Manufacturing Company, Kingsville, Ohio. Colorless liquid, with odor of rose and also of carbolic acid.

3. *Jessop's Local Anæsthetic.* B. J. Pressley, D. D. S., Hammonton, N. J. Colorless liquid, with slight odor of carbolic acid.

4. *Dorsenia, the Brazilian Local Anæsthetic.* Discovered by C. A. Young. From the Dr. C. A. Young Anæsthetic Company, Boston, Mass. Colorless liquid, with distinct odor of camphor.

*Dr. G. Weinmann's Local Anæsthetic.* Brownish-yellow liquid, with odor of creosote or carbolic acid.

6. *Odontunder.* Local anæsthetic for the painless extraction of teeth. Manufactured by the Odontunder Manufacturing Company, Fredonia, N. Y. Colorless liquid, with odor of rose.

7. *Dental Surprise.* Manufactured by the Slocum Manufacturing Company, Holly, Mich. Colorless liquid, with carbolic acid odor.

8. *Dr. E. T. Burr's Local Anæsthetic.* For the painless extraction of teeth. Manufactured by E. E. Barr, dentist; Bowling Green, Ky. Nearly colorless liquid, with odor of peppermint and cloves.

9. *Eureka Anæsthetic.* S. R. Osmun, Morristown, N. J. Colorless liquid, with slight carbolic odor.

10. *Anæsthetic-Obtundent No. 2.* W. Irving Thayer, D. D. S., Williamsburg, Mass. Slightly yellowish liquid; odor of camphor and mixture of essential oils.

All of these were given a careful qualitative examination, and then the total non-volatile matter was determined. Chlorin determinations were next made from which the anhydrous cocain hydrochlorate could be calculated. The pure alkaloid cocain was then extracted with chloroform, the difference between this figure and that obtained from the chlorin determination indicating decomposition products due to the use of commercial cocain salts. The results are summarized in tubular form (see table).

I remain, yours very truly,

SAMUEL P. SADTLER.

### ANALYSIS OF LOCAL ANÆSTHETICS.

| | Non-Volatile Matter. | Anhydrous Cocain Hydrochlorate from Chlorin Determinations. | Alkaloid Extracted by Chloroform. | Other Constituents. |
|---|---|---|---|---|
| 1. Dickson's | 4.06 | 3.90 | 3.27 | Carbolic acid, chloral hydrate. |
| 2. Arophene | 12.92 (Liquid.) | 1.46 | 1.05 | Carbolic acid, chloral, glycerine, oil of rose and probably alcohol. |
| 3. Jessop's | 3.82 | 2.63 | 1.22 | Carbolic acid, oil of rose. |
| 4. Dorsenia | 0.30 | 0.20 | 0.145 | Carbolic acid, camphor, and probably alcohol. |
| 5. Weinmann's | 5.46 | 5.68 | 3.72 | Alcohol, oil of peppermint, brown color, and iodin, indicating aristol possibly. |
| 6. Odontunder | 10.14 (Liquid) | 1.35 | 1.10 | Carbolic acid, glycerin, oil of rose, and probably alcohol. |
| 7. Dental Surprise | 1.37 | 1.46 | 1.16 | Carbolic acid. |
| 8. Barr's | 0.06 | none. | none. | Alcohol solution of oils of peppermint and cloves. |
| 9. Eureka | 3.36 | 3.26 | 1.37 | Carbolic acid and oil of rose. |
| 10. Anæstheto-Obtundent | (Liquid) | 3.39 | 2.61 | Carbolic acid, camphor, glycerin, oils of cinnamon and citronella, and probably alcohol. |

It will be seen from this report that all of the preparations examined, with the exception of Barr's, were found to contain cocain. This exhibit becomes interesting from the fact that in some cases the proprietors either explicitly deny that their preparation contains cocain, or so word their statements as to warrant that inference. In support of this, I quote as follows from a pamphlet entitled "Dorsenia," published by "The Dr. Young Anæsthetic Company":

"To those who are unfamiliar with this remarkable drug we would say that while it is a most reliable local anæsthetic, acting immediately with lasting anæsthetic properties, its composition is *absolutely void* of cocaine, chloral, arsenic, opium, or any poisonous drugs. Thus it may be used on all classes of patients with entire safety.

"The use of Dorsenia in dental surgery has met with such universal success that the medical profession, one by one, are adopting its use in minor surgery as a substitute for cocaine and freezing. Dorsena has so many advantage over cocaine that it at once commends itself to the surgeon."

In the same pamphlet, under the heading, "The Disadvantages of Cocaine, Gas, and Other Vapors, Ether and Chloroform," it says,—

"Cocaine—Action uncertain; must wait ten to fifteen minutes for desired effect; anæsthesia rarely complete. In the majority of cases, after effects, serious toxic symptoms. Many deaths have been reported."

In the same pamphlet, under the title, "The Advantages of Dorsenia," it states that

"It may be used on all classes of patients, the young, the old, the sick, and the healthy, and for the extraction of every tooth in the jaw at one sitting, without danger. No after effects."

And further.

"The value of Dorsenia has been demonstrated before such societies as the First District Dental Society of New York, the Union Convention of Dentists in Boston, Mass., the Dominion Dental Society, Montreal, Canada, etc."

The analysis shows this preparation to contain at least 0.20 per cent. of cocaine hydrochlorate.

In a circular issued by the Arophene Manufacturing Company, Kingville, Ohio, occur the following statements:

"Dentists who value their practice and reputation should beware of the many cocaine preparations now being put upon the market. If you use them trouble may come when you least think, and ruin your business. Use Arophene and be on the safe side.

"The dental profession has already learned much of the new remedy aristol, which is being so thoroughly discussed in all the leading dental and medical journals of the day. This remedy enters largely into the composition of Arophene, which alone should be a guarantee that it is the latest as well as the safest and best local anæsthetic on the market."

The analysis shows entire absence of aristol.

"Arophene will positively destroy all sense of pain, and cause no after trouble. It is applied to the gums, so that there is no more need of inhaling poisonous drugs, such as chloroform, ether, gas, etc., thereby risking one's life, or at least impairing their health. It can be used on any one, even those suffering with heart-disease, lung trouble, or nervous debility, whether young or old.

"You are aware that the market is flooded with so-called local anæsthetics. Many of these are imitations of Arophene, trying to follow in our footsteps only to fall far short of their desires. Do not be deceived. Do not trifle with those cheap cocaine preparations, for when you least expect it, you may ruin your practice and your professional standing.

"No antidote is necessary when using Arophene, more than a swallow of cold water, or dilute alcohol or other stimulants, and fresh air. Be pleasing, and make your patient feel cordial and pleasant as possible. Apply Arophene to the gums by means of cotton, then insert the point of a fine hypodermic needle in the tissues surrounding the teeth to be extracted, at an angle, going deeper as the gums turn white, using alike on each side of teeth. Wait about two minutes and extract, going down low enough to assure the teeth not to break. Have no fear in using Arophene. It is the safest anæsthetic ever used."

The foregoing suggestions for the relief of unpleasant symptoms are those which, as you are aware, are used in cocain-poisoning, and the analysis shows the presence of 1.46 per cent. of cocain.

In the circular of "Directions for the Use of Thayer's Anæstheto Obtundent" occurs the following statement:

"The proprietor wishes it distinctly understood that his Anæstheto Obtundents are not death-producing compounds, hence the benumbing qualities are recommended for *five minutes* only. They will act sharply for that time; the anæsthesia commences in ten seconds on soft tissues, and increases, which is not the case with cocaine."

The analysis of this preparation shows 3.39 per cent. cocain hydrochlorate.

In the circular published by the Dickson Manufacturing Company, Sharon, Pa., entitled "Special Directions for Dickson's Improved Anæsthetic," it states,—

"Occasionally a patient may complain of faint feeling or slight sickness at stomach; to relieve this, give twenty drops of aromatic spirits of ammonia in a little water."

A very good antidote to poisoning by cocain, of which there is 3.90 per cent. in this preparation.

Viewed from a standpoint of a knowledge of their composition, the claims of the proprietors of these nostrums, as set forth in their respective circulars, make interesting reading, with which you are all no doubt quite familiar, but enough has been here quoted to show at least one phase of its character.

The question then arises: Is the use of cocain or its salts in the manner and for the purposes set forth a legitimate procedure under conditions where the operator is not aware of the fact that he is using cocain? Let us see what the status of the admitted and recognized action of cocain is at present, and whether it is regarded as a uniformly safe drug to use, even with a full knowledge of the rationale of its physiological action and consequent command of the situation which such a knowledge entails, whereby any untoward action of the drug which threatens life may be promptly and intelligently combated.

The deaths which have occurred from cocain administration are quite numerous; current medical and dental literature for the past few years has contained frequent reports of such fatalities. Moreover, the dosage under which fatal casualties have occurred has varied to such an extent that no fixed limit can be positively placed for a maximum safe dose for this drug. It has been shown conclusively that one is quite likely to frequently meet temperamental idiosyncrasies in individual cases, where even minute doses of cocain may produce the most alarming symptoms. In support of this, I quote the following communication from Dr. Mattison, medical director of the Brooklyn Home for Habitues, published in the Philadelphia *Times and Register*, October 15, 1892. The article is in reply to a statement in a previous issue by the editor of that journal. Dr. W. F. Waugh, respecting syncope following the local use of cocain in two patients, of which he says, "They were persons who could be expected to faint on any suitable occasion, and my impression is that his is true in all, or nearly all, reported cases."

Dr. Mattison, however, thinks differently; he says,—

"In three papers by myself,—'Cocain Dosage and Cocain Addiction,' *London Lancet*, May 23, 1887; 'Cocain Toxæmia,' *La Tribune Medical*, Paris, January 1, 1888; and 'Cocain Poisoning,' *Medical and Surgical Reporter*, October 24, 1891,—more than two hundred cases of poisoning by cocain, including thirteen deaths, are reported. In a fourth paper—soon to appear—more deaths and other toxic cases are cited, making a record that medical men should ever have in mind when using this valued but dangerous—sometimes—drug.

"I think your 'impression' that 'all, or nearly all, the reported cases were persons who could be expected to faint on any suitable occasion,' a mistaken one. It will never do to presume that a patient who, by virtue of robust build, is not likely to faint, will be proof against the untoward effect of cocain, for he who puts such an opinion in practice will run large risk of 'coming to grief.'

"Many of the patients noted in my papers were not subject to syncope, and in the latest reported fatal case in this country,—a recent occurrence in Bellevue Hospital, —a strong, healthy man, who was given a four per cent. solution by urethral injection, died in less than four minutes!

"It may not be amiss to repeat the conclusions of my studies in cocain-toxæmia:

"1. Cocain may be toxic.

"2. This effect is not rare.

"3. There is a lethal dose of cocaine.

"4. This dose is uncertain.

"5. Dangerous or deadly results may follow doses usually deemed safe.

"6. Toxic effects may be the sequence of doses large or small, in patients youny or old, the feeble or the strong.

"7. The danger, near and remote, is greatest when given under the skin.

"8. Cardiac or renal weakness increases the risk.

"9. Purity of drug will not exempt from ill result.

"10. Caution is needful under all conditions.

"11. Reclus's method, Corning's device, or Esmarch's bandage should be used when injecting.

"12. Nitrite of amyl, hypodermic morphine, hypodermic ether, alcohol, ammonia, and caffein should be at command."

In a paper read at the tenth annual meeting of the American Rhinological Association, at Indianapolis, September 22, 1892, on "The Uses and Abuses of Cocain," Dr. Arthur G. Hobbs, of Atlanta, Ga., says,—

"Since I received my first twenty grains of the hydrochlorate of cocain (a part of the first two-drachm package that arrived in New York), I have used many ounces; perhaps, altogether, by myself and assistants, almost as many pounds as my original package contained grains.

"It will not be amiss for me to say, in the beginning, that I now have very different opinions of its uses from those I entertained during the first years of its advent.

"The general symptoms are, everything else being equal, much oftener present in proportion to the area of membrane surface involved than in proportion to the strength of the solution applied. Hence, sprays of a weak solution are liable to produce toxic symptoms, when much stronger solutions can be applied with a saturated cotton probe or wad, to a limited area, almost with impunity.

"The constitutional effects of cocain are manifested by a feeling of faintness,—it may be even to the loss of consciousness,—a trembling of the limbs, a pallor of the face, any or all of which may appear in one minute or in fifteen minutes after the application to a large surface. Again, these symptoms may be produced by a small quantity of a one per cent. solution in one individual and not in another; even if in the latter both the strength and quantity be ten times multiplied. I have never reached a conclusion as to the average amount of cocain that is necessary to produce the constitutional symptoms; neither do I ever know, the first time I use it, how to estimate the individual's susceptibilities.

"Every individual seems to be a law unto himself as to the quantity necessary in his own case to accomplish complete local anæsthesia; and even in the same person the amount will vary at different times, with no apparent cause. Under similar circumstances I find it impossible to reach a complete local anæsthesia in some, short of the toxic symptoms; when in others one-tenth of the amount and one-tenth of the time will render the application of a galvano-cautery absolutely painless."

We have now shown by the record of analyses that the nostrums so far examined, with a single exception (Barr's), depend for their action upon the cocain they contain, and we have adduced expert medical testimony to show that the introduction of cocain into the circulation is under certain circumstances attended with grave danger to life; furthermore, that the conditions under which cocain may be safely introduced into the circulation are not well known, or only incompletely made out. It requires no further argument to support the assertion that the use of a drug which is known to be possessed of dangerous activities becomes culpable when its identity is disguised in the form of a nostrum, leaving the administrator absolutely handicapped by reason of his ignorance of the nature of the preparation and of the rational restorative treatment which should be applied in case of threatened danger to the life of his patient. His only resource in such event would be to treat the case on general principles, or to rely on the meager directions furnished by the quack who compounded the nostrum.

The treatment of cocain syncope is a sufficiently grave and difficult matter when its causation is perfectly known, but when it occurs from cocain *incognito* it is a combat in the dark, with the chances mostly in favor of the enemy.

From the ethical standpoint, the use of this class of preparations appears to be wrong, not only for the general reason that the use of all nostrums is wrong, but in addition because they may become a menace to life. From a purely commercial standpoint the traffic in these preparations is, to put it mildly, unjust.

The present market price of cocain hydrochlorate is about five dollars per ounce, wholesale. A four per cent. solution is therefore worth twenty cents per ounce. The record of analyses of the nine preparations which I have presented to you this evening show amounts of cocain varying from .20 to 5.68 per cent., which makes an average of 2.59 per cent., representing an actual average value of (in round numbers) thirteen cents for each ounce of the nostrum, and this is sold to the dental practitioner at an average price of one dollar and fifty cents per ounce.

I have left out of the computation the other ingredients, as they are of such slight value as to have no material effect upon the cost. In the single preparation here reported which contains no cocain, the compound consists of one ounce of alcohol containing a few drops each of oil of peppermint and oil of cloves, and this preparation having an actual drug value of less than five cents, is sold at the retail price of one dollar per ounce. It is probably physiologically harmless, and is therefore not to be included in the special ethical considerations applicable to those preparations containing cocain. It is simply a fair example of the usual type of quack nostrums, in which the consumer pays an exorbitant price for a trifling drug value.

My object in bringing these data before you has been to call your attention not only to what I regard as a breach of the spirit of our Code of Ethics, but a condition of affairs which seems to me to constitute a real danger to the dental profession and the community at large, and it is in the spirit of this belief that I commend these statements to your consideration.—*Dental Cosmos.*

